# NGF/ERK signaling-mediated epigenetic regulation of neuropathic pain in the cerebrospinal fluid-contacting nucleus

**DOI:** 10.3389/fneur.2025.1641450

**Published:** 2025-10-29

**Authors:** Guangling Li, Chao Wang

**Affiliations:** ^1^Department of Anesthesiology, Affiliated Hospital of Jiangnan University, Wuxi, Jiangsu, China; ^2^Department of Gastrointestinal Surgery, Affiliated Hospital of Jiangnan University, Wuxi, Jiangsu, China

**Keywords:** CSF-CN, NGF, ERK1/2, acetylated histone, neuropathic pain, CCI

## Abstract

**Objective:**

Neuropathic pain (NP) is a debilitating condition that is associated with multiple molecular alterations in the nervous system. This is the first study to demonstrate the epigenetic regulatory function of nerve growth factor (NGF), mediated through the extracellular signal-regulated kinase (ERK) signaling pathway in the cerebrospinal fluid-contacting nucleus (CSF-CN), in relation to NP. The objective of this study was to characterize the role of NGF in this specific brain region and its downstream effects on histone acetylation in the chronic constriction injury (CCI) rat model.

**Methods:**

A rat model of NP was established using CCI. The study investigated the expression of NGF and its impact on pain pathways. NGF levels in the CSF-CN were assessed, and the effects of NGF on neuropathic pain were evaluated by administering NGF antibodies and ERK antagonists. Immunohistochemistry and Western blotting were used to measure key molecular markers, including p-ERK, THMA, and acetylated histone H3K56. Statistical analysis was performed, with significance set at a *p*-value of < 0.05.

**Results:**

CI induced significant upregulation of NGF in the CSF-CN. Treatment with NGF antibodies alleviated NP symptoms and reduced p-ERK levels in the CSF-CN. ERK antagonists further diminished thermal hyperalgesia and mechanical allodynia (THMA) and decreased the expression of acetylated histone H3K56.

**Conclusion:**

This is the first study to identify an NGF/ERK/ acetylated histone H3 (Ac-H3) signaling axis in the CSF-CN as a central mediator of neuropathic pain through epigenetic modulation. Our results suggest that targeting this novel signaling pathway may provide new therapeutic strategies for managing NP.

## 1 Introduction

Neuropathic pain (NP) is a complex and often debilitating condition resulting from damage or disease affecting the nervous system. Unlike nociceptive pain, which arises from tissue damage, NP is characterized by the abnormal processing of sensory signals within the nervous system, leading to persistent pain even in the absence of clear injury or inflammation (1). The pathophysiology of NP involves complex changes at both peripheral and central levels, including altered neuronal excitability, neurotransmitter imbalances, and neuroinflammation (2).

Central sensory fiber-related neuroinflammation plays a critical role in NP by modulating the sensory pathways involved in pain perception. Central sensory fiber-related neuroinflammation encompasses various cellular and molecular components within the central nervous system (CNS) that contribute to the amplification and maintenance of NP (3). The anatomical locations of the Central sensory fiber-related neuroinflammation, specifically the dorsal horn of the spinal cord and brainstem, are pivotal in integrating and processing nociceptive signals. The cellular composition of these regions includes a range of neurons and glial cells that are involved in both the initiation and modulation of pain responses (4).

A significant pathway associated with NP is the nerve growth factor (NGF)/extracellular signal-regulated kinase (ERK)/acetylated histone H3 (Ac-H3) pathway. NGF, a key neurotrophin, activates the ERK signaling cascade, which subsequently leads to the acetylation of histone H3 at lysine 56 (H3K56) (5). This acetylation modulates gene expression related to pain and inflammation, thereby contributing to the persistence and intensity of NP (6). Understanding the NGF/ERK/Ac-H3 pathway highlights the complex molecular mechanisms underlying NP and underscores potential targets for therapeutic intervention.

To the best of our knowledge, this study is the first to identify the role of the NGF/ERK signaling pathway in the cerebrospinal fluid-contacting nucleus (CSF-CN) as a site of epigenetic regulation in neuropathic pain, thereby adding a new anatomical consideration for neuromodulation of both acute and chronic processes of neuropathic pain. In addition, this focus on the CSF-CN extends beyond classical pain pathways and introduces a supraspinal structure that may influence NP through histone acetylation. The chronic constriction injury (CCI) model was selected because it is a reproducible and widely accepted model of neuropathic pain, which is known to induce robust NGF upregulation and behavioral hypersensitivity (7, 8), thereby providing a reliable platform for investigating supraspinal mechanisms.

Despite significant progress, research on NP faces considerable challenges. One major issue is the heterogeneous nature of NP, which can vary greatly between individuals and conditions, complicating both diagnosis and treatment (9). Furthermore, current animal models often fail to fully replicate the human experience of NP, leading to limitations in the applicability of findings to clinical settings (10). Moreover, the lack of a comprehensive understanding of the interactions between peripheral and central mechanisms and the role of genetic and environmental factors hinders the development of effective treatments. Addressing these challenges requires a multidisciplinary approach integrating advances in molecular biology, neuroimaging, and patient-specific research to refine existing models and therapeutic strategies. By advancing our knowledge of the underlying mechanisms and overcoming these research barriers, we can develop more targeted and effective interventions for managing NP.

## 2 Materials and methods

### 2.1 Animals

Our study received approval from the International Association for the Study of Pain and the Committee for the Ethical Use of Laboratory Animals, the Affiliated Hospital of Jiangnan University. The animal study protocol was approved by the Ethics Committee of the Affiliated Hospital of Jiangnan University (Approval No JNU-AE-2023-014). Male Sprague Dawley SPF rats (250 ± 50 g) were obtained from the Experimental Animal Center at Xuzhou Medical College. The animals were housed under controlled climate and lighting conditions (23 ± 1 °C, 12/12-h dark/light cycle, with lights on at 8:00 a.m.) for at least 1 week before testing.

#### 2.1.1 Neuropathic pain model

The CCI model developed by Bennett et al. was used in this study (32). The animals were anesthetized with 10% chloral hydrate. The left sciatic nerve was isolated from the surrounding tissue. Four loose ligatures were placed around the nerve at an interval of 1 mm using 4–0 braided silk thread. The incision was not made in layers. In sham-operated rats, the same procedure was performed without the ligation of the sciatic nerve.

#### 2.1.2 Behavioral analysis

All behavioral experiments were conducted under controlled conditions. The rats were acclimated to the bottom cages for 30 min before the tests, which were conducted 1 day before CCI surgery and on days 1, 3, 7, and 14 after CCI.

##### 2.1.2.1 Mechanical allodynia

Mechanical hyperalgesia was assessed by applying a series of discrete forces (0.16, 0.4, 0.6, 1, 1.4, 2, 4, 6, 8, 10, and 15 g) to the sole of the left hind paw. Each force was applied for up to 6 seconds, or until a withdrawal reaction or paw licking occurred.

##### 2.1.2.2 Heat hypersensitivity test

The rats were placed in a Plexiglass enclosure on the glass surface of a thermal device for 30 min before evaluating thermal withdrawal latency (TWL). A mobile radiant heat source was directed at the hind paw, and TWL was recorded using a digital timer. The thermal stimulus was mediated to produce a mean baseline paw withdrawal latency of approximately 10s in the sham-operated or natural hind paw. A cutoff time of 20 s was set to prevent tissue injury.

#### 2.1.3 Drug administration

The ERK1/2 antagonist U0126 (Sigma-Aldrich, Cat# V1121) was dissolved in dimethyl sulfoxide (DMSO). The NGF neutralizing antibody (Abcam, Cat# ab6199, RRID: AB_305320) was prepared in phosphate-buffered saline (PBS). The rats were anesthetized with 10% chloral hydrate (300 mg/kg, i.p.) 6 days post-CCI and then placed in a stereotaxic frame. Either U0126 (10 μg/5 μL) or the NGF antibody (10 μg/5 μL) was administered as a single intracerebroventricular (i.c.v.) injection at stereotaxic coordinates (Bregma: −1.2 ± 0.4 mm, depth: 3.2 ± 0.4 mm, lateral: 1.4 ± 0.2 mm). Behavioral tests (Thermal Plantar, von Frey) were conducted 24 h after drug administration.

To investigate the ERK–histone acetylation connection, we included a histone acetyltransferase (HAT) inhibitor group and a combination group: C646 (10 μg/5 μL, i.c.v.) alone and U0126 (10 μg/5 μL) + C646 (10 μg/5 μL), administered 6 days post-CCI at the same stereotaxic coordinates; vehicle-matched DMSO/PBS was used accordingly. Behavioral testing was performed 24 h after injection, as described above.

### 2.2 Immunofluorescence procedures

A 3 μL injection of 30% CB-HRP (Sigma-Aldrich, St. Louis, MO, USA) was administered into the lateral ventricle (LV) using the stereotaxic coordinates described above to mark the CSF-CN. Furthermore, 48 h later, the rats were deeply anesthetized with 10% chloral hydrate and underwent transcardial perfusion with 0.01 M PBS followed by 4% paraformaldehyde in 0.1 M phosphate buffer for fixation. The brainstem was immediately removed and post-fixed for 4–6 h at 4 °C in the same fixative, and the tissue was dehydrated in 30% sucrose in 0.1 M PB until it sank to the bottom. The brainstem was embedded in OCT at −20 °C and sectioned using a cryostat at 35 μm within the transverse plane. The slices were rinsed with 0.3% Triton X-100 for 15 min, blocked with donkey serum for 1 h at room temperature, and subsequently incubated with goat anti-CB and anti-rabbit NGF (1:100, Abcam Cambridge, UK), anti-rabbit Ac-H3K56 (1:200, Abcam), or anti-rabbit p-ERK1/2 for 24 h. Afterward, the sections were incubated for 1 h with FITC-labeled donkey anti-goat and FITC-labeled donkey anti-rabbit antibodies. Ultimately, the sections were washed, mounted, fixed onto slides, and stored at −20 °C in the dark. Each section was then examined using a confocal laser microscope.

### 2.3 Western blotting

After the behavioral trial, the CSF-CN region of the brain was separated and stored at −80 °C. Proteins were extracted from the CSF-CN using RIPA lysis buffer. The protein specimens were isolated using an 8% SDS-polyacrylamide gel and then electroblotted, which were subsequently blocked with the added 5% skim milk in Tris-buffered saline (TBS) with Tween for 1 h at room temperature. They were then incubated overnight with primary antibodies at 4 °C. The antibodies used were anti-rabbit Ac-H3K56, anti-rabbit H3 (1:1000, CST), and anti-mouse *β*-actin (1:1000, Abcam). After three rinses in TBS with Tween, the membranes were deeply probed with the appropriate secondary anti-rabbit IgG antibody (1:1000, Sigma).

In addition to Ac-H3K56 and H3, the membranes were also probed for p-ERK1/2 and total ERK1/2. Semiquantitative densitometry was performed using p-ERK/ERK and Ac-H3K56/H3 ratios (*n* = 6 per group).

The ImageJ software was used to perform semi-quantitative analysis of the Western blot grayscale images for Ac-H3K56, H3, and *β*-actin.

### 2.4 Statistics

Statistical analyses were performed using GraphPad Prism 9.0 (GraphPad Software Inc., USA). Data were presented as mean + SEM. Two-way repeated measures ANOVA was used to analyze the behavioral tests (mechanical withdrawal threshold (MWT) and TWL) for group and time effects. Ryan’s test was utilized as a *post hoc* analysis to compare the treatment and control groups. *p*-values of < 0.05 were considered statistically significant.

## 3 Results

### 3.1 Development of thermal hyperalgesia and mechanical allodynia

**Figure 1:**
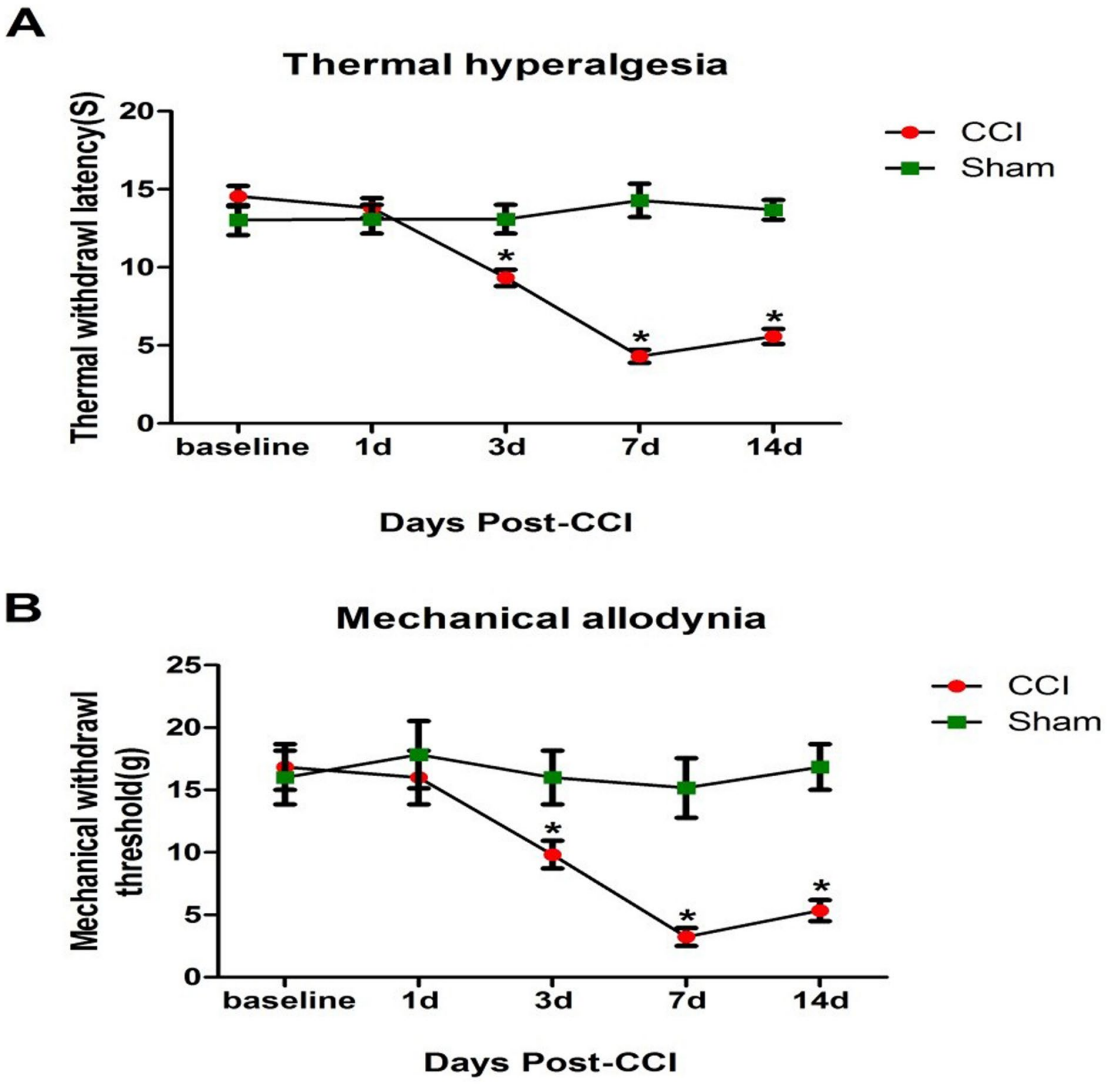
Evaluation of behavioral trials. The rats underwent CCI or sham surgery, and behavioral assessments were conducted at baseline and post-surgery using TWL and MWT tests. **(A)** Time course of thermal withdrawal latency (TWL) values following CCI surgery. **(B)** Time course of mechanical withdrawal threshold (MWT) values following CCI surgery. *p < 0.05, compared to the sham group.

Thermal Plantar and von Frey tests were used to determine the mechanical withdrawal threshold (MWT) and thermal withdrawal latency (TWL). Compared to baseline, MWT values significantly declined from day 3 post-chronic constriction injury (CCI) surgery until day 14, reaching a minimum on day 7 (Figure 1A). Similarly, TWL values (Figure 1B) showed that thermal hyperalgesia (TH) and mechanical allodynia (MA) began on day 3 post-CCI and remained stable until day 14, peaking on day 7.

### 3.2 Distribution of Ac-H3K56 in the rat brain parenchyma

**Figure 2:**
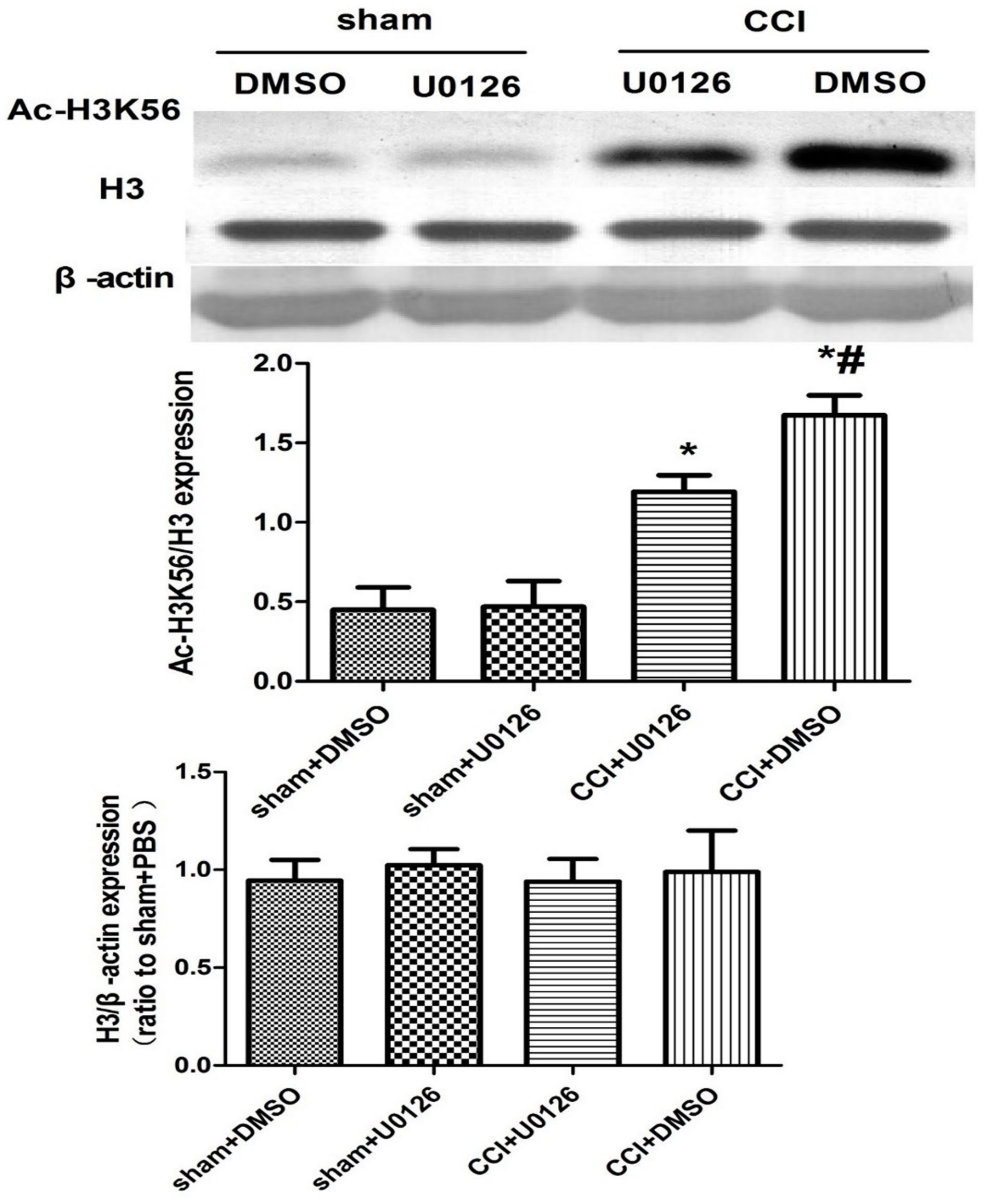
Double labeling of cerebrospinal fluid-contacting nucleus (CSF-CN) neurons using CB-HRP and Ac-H3K56 immunofluorescence in the healthy rats. **(A)** CB-HRP-positive neurons (red). **(B)** Ac-H3-positive neurons (green). **(C)** Dual-labeled neurons (yellow). Scale bar = 100 μm.

CB-HRP-labeled neurons (red) in the ventral periaqueductal gray (PAG) of the brainstem constitute the cerebrospinal fluid-contacting nucleus (CSF-CN). Dual-labeled immunofluorescence revealed the presence of Ac-H3K56 in the CSF-CN, with Ac-H3K56-immunoreactive neurons (green) located near the midline of the ventral mesencephalic aqueduct (Aq). Dual labeling (yellow) verified the presence of Ac-H3K56 (Figure 2).

### 3.3 Attenuation of allodynia and hyperalgesia following intracerebroventricular injection of the NGF antibody

**Figure 3:**
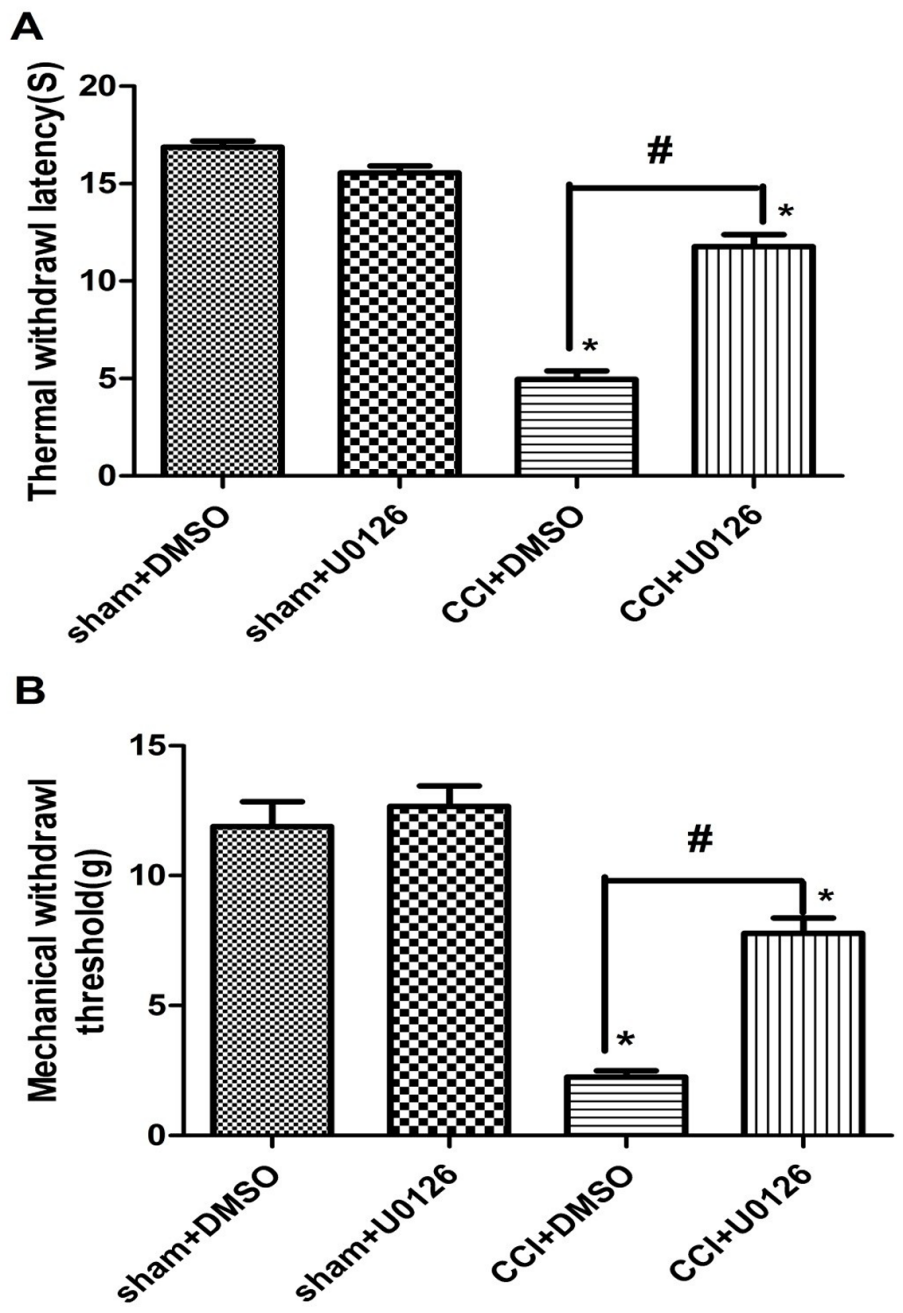
The NGF antibody alleviated thermal hyperalgesia and mechanical allodynia after CCI surgery. The rats received a single intracerebroventricular (i.c.v.) injection of the NGF-neutralizing antibody (10 μg) 6 days after CCI surgery. Behavioral tests were performed 24 h later. The NGF antibody significantly increased both the MWT and TWL compared to the CCI + DMSO controls, indicating attenuation of pain behaviors. *p* < 0.05 versus sham + DMSO; #p < 0.05 versus CCI + DMSO, *n* = 6.

In the CCI rats, both the mechanical withdrawal threshold (MWT) and thermal withdrawal latency (TWL) were assessed 24 h after intracerebroventricular injection of the NGF antibody (10 μg/i.c.v.). The rats treated with the NGF antibody showed a highly significant alleviation of neuropathic pain (NP) symptoms, as evidenced by increased MWT and TWL, indicating a significant reduction in mechanical allodynia (MA) and thermal hyperalgesia (TH), respectively (Figure 3).

### 3.4 Lateral intracerebroventricular injection of the NGF antibody decreased Ac-H3K56 expression in the CCI rats

**Figure 4:**
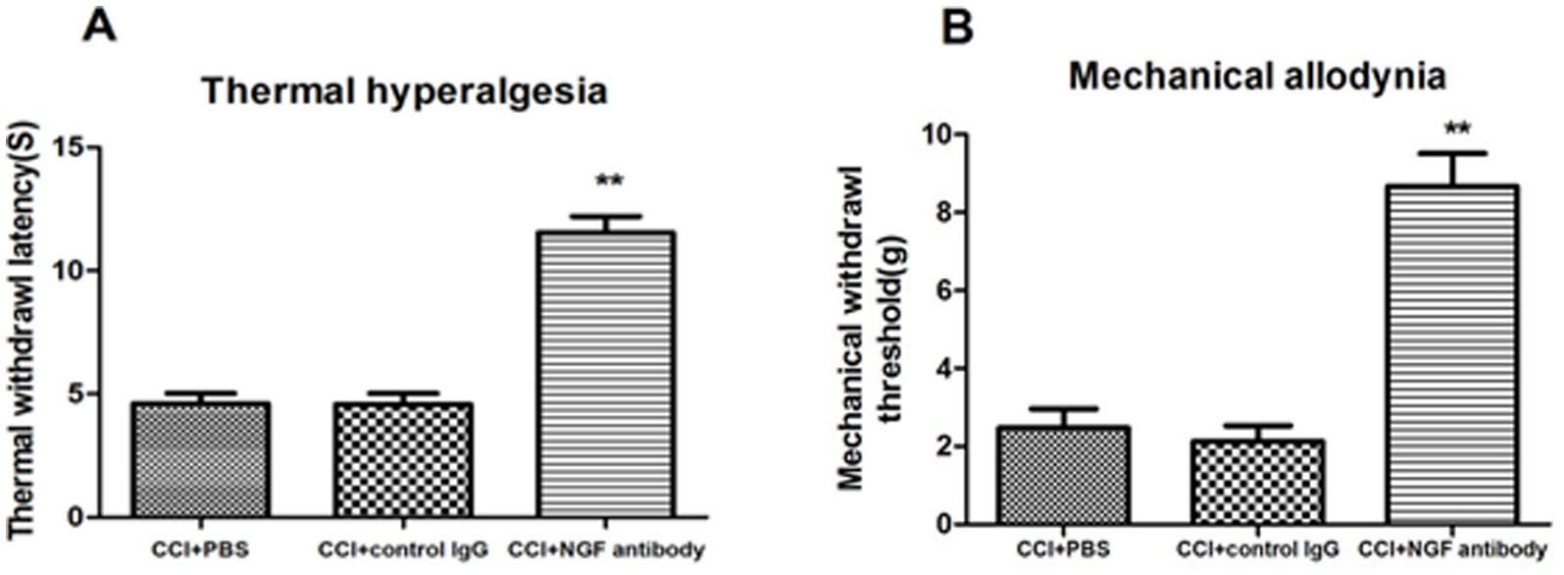
Western blot analysis of Ac-H3K56 expression after NGF antibody administration. Protein extracts from the CSF-CN were collected 24 h after NGF antibody (10 μg, i.c.v.) or PBS injection. Western blotting was performed using an anti-Ac-H3K56 antibody, with *β*-actin as a loading control. Quantification showed significant downregulation of Ac-H3K56 in the NGF antibody-treated rats. *p* < 0.05 versus sham + PBS; #p < 0.05 versus CCI + PBS, *n* = 6.

The NGF antibody affected the expression of p-ERK, suggesting that NGF triggers epigenetic alterations of histone H3 tails and influences pain-related gene expression through the ERK pathway. The NGF antibody significantly downregulated Ac-H3K56 expression in the CSF-CN 24 h post-injection, implicating the ERK/Ac-H3 pathway in CCI-associated chronic pain (Figure 4).

### 3.5 Intracerebroventricular U0126 attenuates allodynia and hyperalgesia and downregulates Ac-H3K56 expression in CCI Rats

**Figure 5:**
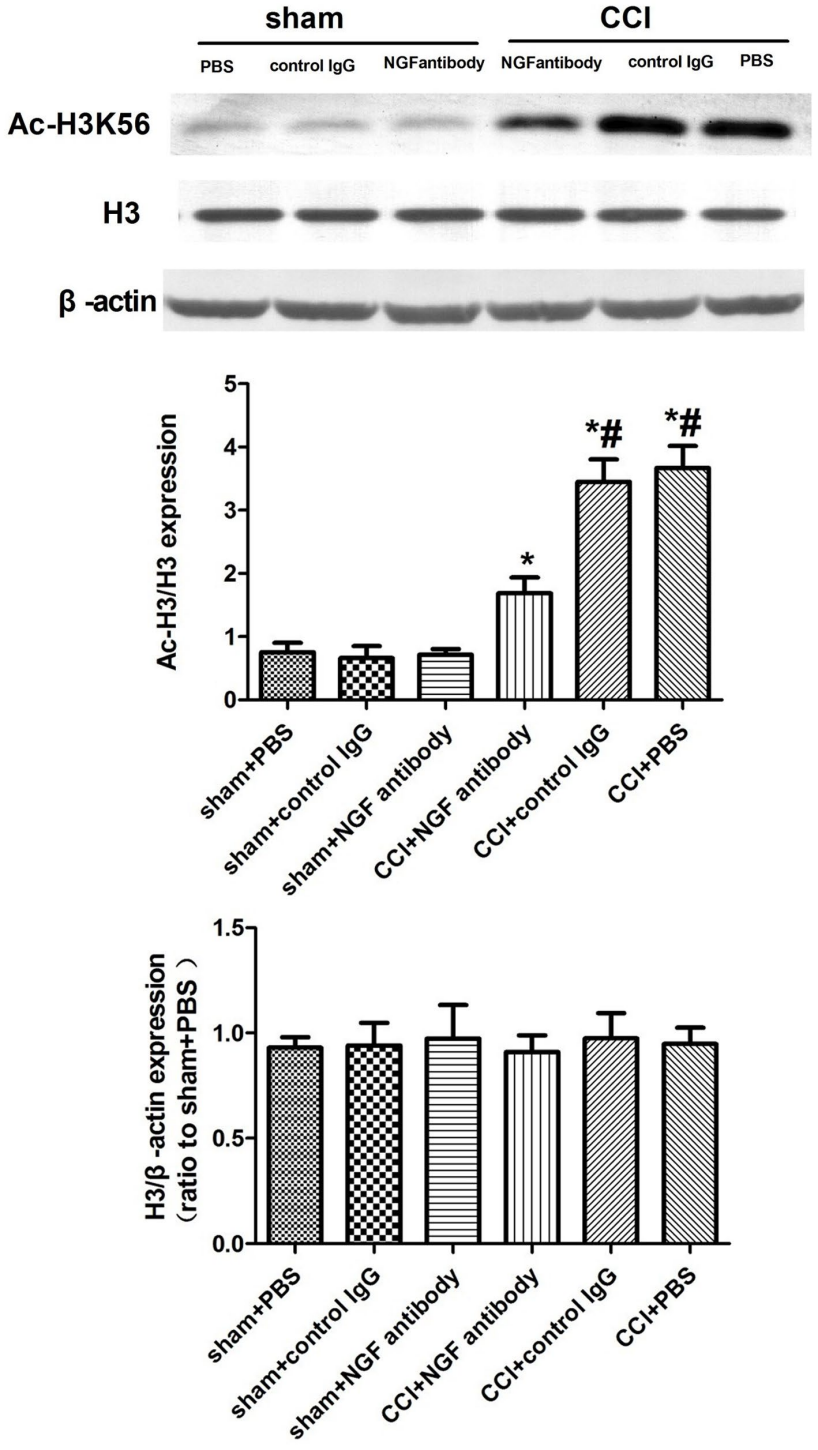
ERK1/2 inhibitor (U0126) attenuated neuropathic pain behaviors after CCI surgery. The rats received a single i.c.v. injection of U0126 (10 μg) 6 days after CCI surgery. Behavioral assessments were conducted 24 h later. Both TWL and MWT were significantly increased in the U0126-treated rats compared to the CCI + DMSO controls, indicating that ERK inhibition alleviates thermal hyperalgesia and mechanical allodynia. p < 0.05 versus sham + DMSO; #p < 0.05 versus CCI + DMSO, *n* = 6.

**Figure 6:**
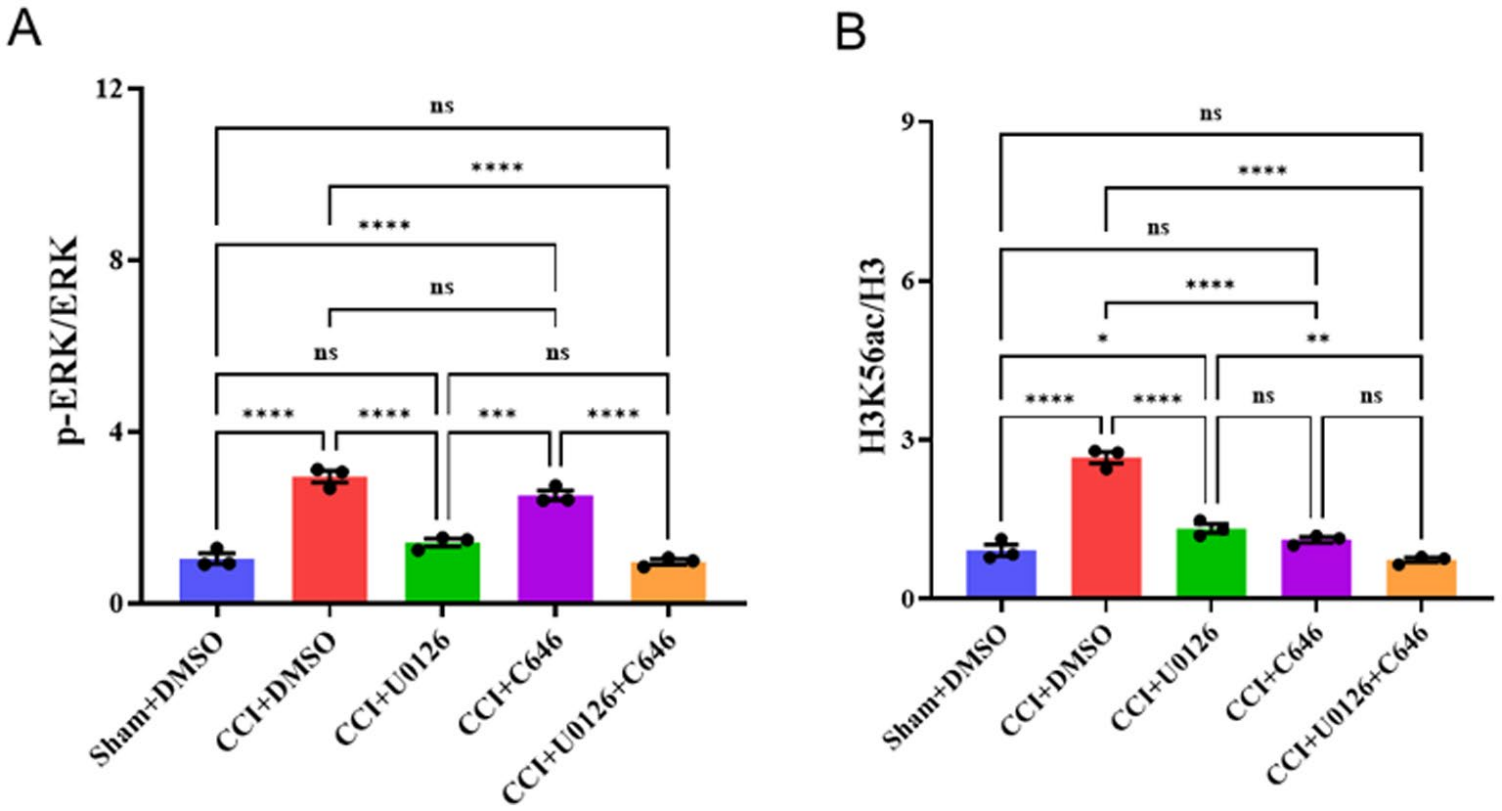
Western blot analysis of Ac-H3K56 expression after ERK inhibition. CSF-CN tissue was harvested 24 h after i.c.v. injection of U0126 (10 μg). Western blotting using an anti-Ac-H3K56 antibody revealed that ERK inhibition significantly reduced H3K56 acetylation compared to the CCI + DMSO controls. p < 0.05 versus sham + DMSO; #p < 0.05 versus CCI + DMSO, *n* = 6.

The ERK inhibitor U0126 was used to assess the role of NGF/ERK signaling in histone H3 acetylation. In addition, 24 h after U0126 (10 μg/i.c.v.) administration, CCI-induced pain was significantly attenuated (Figure 5) and Ac-H3K56 expression was downregulated (Figure 6).

### 3.6 ERK activation drives H3K56 acetylation after CCI: pharmacologic dissociation by U0126 and C646

**Figure 7:**
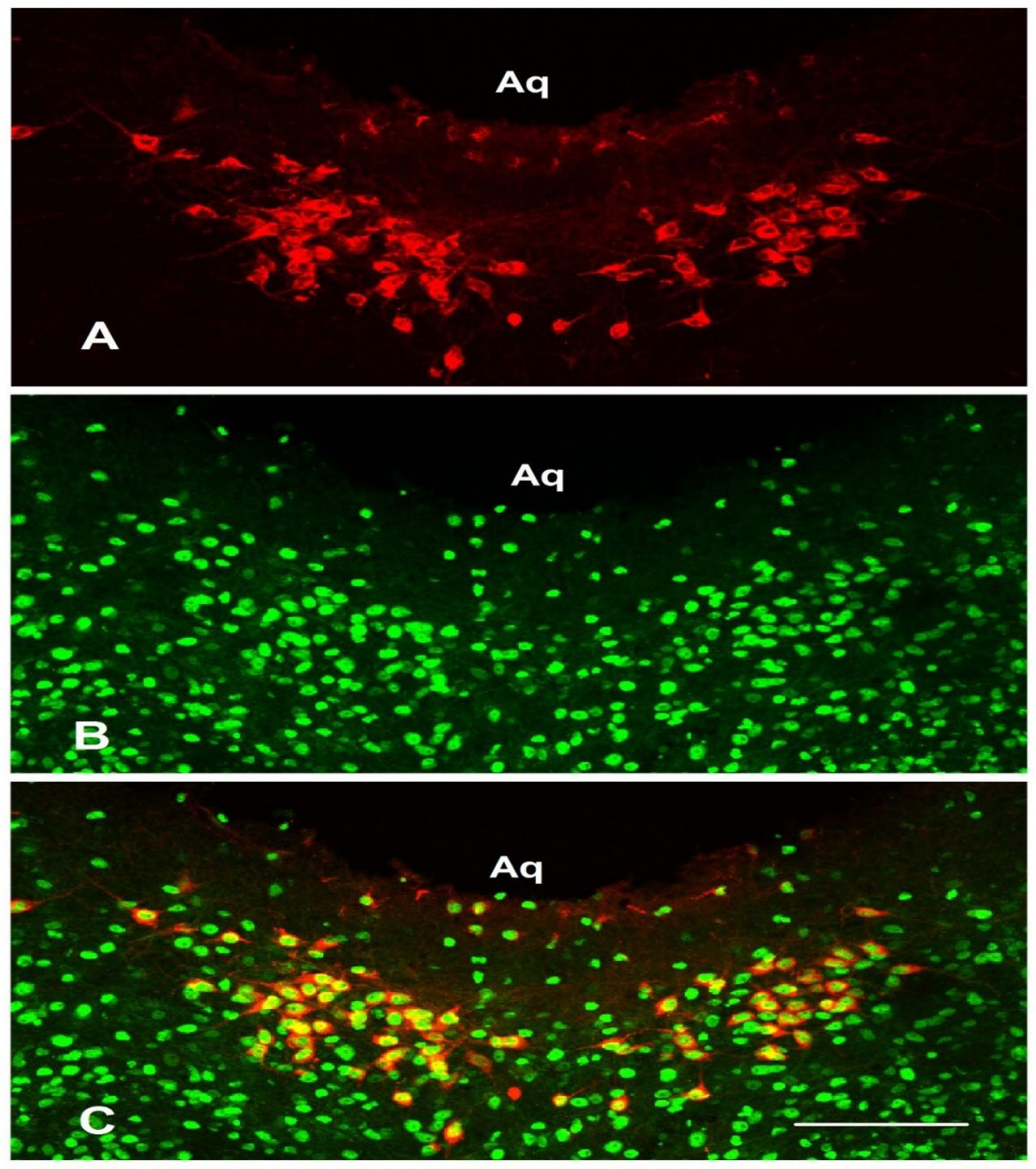
ERK–histone acetylation linkage in the CSF-CN after CCI. **(A)** The p-ERK/ERK ratio in the sham + DMSO, CCI + DMSO, CCI + U0126, CCI + C646, and CCI + U0126 + C646 groups. CCI increased p-ERK; U0126 normalized p-ERK; C646 showed no effect; U0126 + C646 was not different from U0126 alone. **(B)** The Ac-H3K56/H3 ratio in the same groups. CCI increased Ac-H3K56; both U0126 and C646 reduced Ac-H3K56, with no further reduction observed when combined. Data are mean ± SEM; *n* = 6; two-way RM-ANOVA with Ryan’s *post hoc*; ****p < 0.0001, **p < 0.01; ns, not significant.

CCI markedly increased the p-ERK/ERK ratio in CSF-CN tissue compared to sham + DMSO. Intracerebroventricular administration of U0126 (10 μg) normalized the p-ERK/ERK ratio to near-sham levels, whereas the histone acetyltransferase inhibitor C646 (10 μg) did not alter the p-ERK/ERK ratio relative to the CCI + vehicle; the U0126 + C646 combination did not differ from U0126 alone (Supplementary Figure S1). In parallel, CCI robustly increased the Ac-H3K56/H3 ratio. Both U0126 and C646 significantly reduced Ac-H3K56/H3 compared to CCI + vehicle, and the combination produced no further reduction beyond that achieved by either agent alone (Figure 7). These pharmacological dissociations indicate that ERK activation occurs upstream of the histone lysine acetylation change after CCI, while HAT activity is permissive at the acetylation step. Together, the data established a directional ERK → H3K56ac axis in the CSF-CN (*n* = 6 per group; two-way repeated-measures ANOVA with Ryan’s *post hoc* test; ****p < 0.0001, **p < 0.01 as indicated; ns, not significant).

## 4 Discussion

Our study presents new opportunities for understanding the epigenetic landscape of neuropathic pain (NP), highlighting the role of nerve growth factor (NGF) within the NGF/ERK/AcH3K56 signaling pathway. We showed that NGF, which was increased in the cerebrospinal fluid-contacting nucleus (CSF-CN) after chronic constriction injury (CCI), appears to modulate NP: NGF antibody treatment effectively alleviated thermal hyperalgesia and mechanical allodynia while also reducing the expression of acetylated histone H3 at lysine 56 (Ac-H3K56). Furthermore, our results indicated that NGF antibody treatment reduced phosphorylated ERK (p-ERK) expression, suggesting that NGF was primarily acting to promote ERK signaling. This conclusion is also supported by experiments using the ERK antagonist U0126, which decreased both Ac-H3K56 levels and pain behavior. However, due to the lack of direct biochemical validation of NGF-induced ERK phosphorylation in isolated systems, this pathway should be interpreted with caution and confirmed in future studies investigating the mechanism. The mechanistic basis of NGF/ERK-mediated H3K56 acetylation may involve recruitment of histone acetyltransferases, such as CBP/p300, which are known downstream effectors of ERK signaling (11, 12). These enzymes preferentially acetylate lysine residues on histone H3, including H3K56, a modification that is strongly associated with chromatin relaxation and transcription of inflammation-related genes. The selective elevation of Ac-H3K56 in the CSF-CN observed in our study suggests that this epigenetic mark may serve as a critical regulator of pain-related gene expression. However, we did not examine other lysine acetylation sites on histone H3 in this study; therefore, we cannot exclude the possibility that additional residues are also involved. A more definitive demonstration of selectivity will require experiments using histone acetyltransferase inhibitors (such as C646), alone or in combination with ERK inhibition, which we propose as an important future direction. Nevertheless, further biochemical studies are necessary to delineate whether additional histone sites or other HATs are involved and to clarify how ERK inhibition specifically reduces H3K56 acetylation after CCI. In conclusion, to the best of our knowledge, this study is the first to show that NGF modulates neuropathic pain through epigenetic regulation in the CSF-CN, a supraspinal structure that has not been extensively explored in the context of pain signaling. This novel experiment provides evidence to support a new experimental mechanistic model of NP with supraspinal NGF signaling driving NP via chromatin remodeling.

Epigenetic regulation, including histone modifications such as acetylation, plays a crucial role in the accessibility of transcriptional machinery to DNA, thereby influencing the gene expression (13, 14). Histone acetylation, in particular, reduces the electrostatic interaction between DNA and histone proteins, leading to a more relaxed chromatin structure and enhanced gene transcription (6). Recent studies have elucidated the role of histone acetylation in NP. For example, in CCI rat models, elevated expression of histone acetyltransferases (HATs), such as CBP and p300, was observed, which correlated with increased acetylation of histones H3 and H4 (15). Inhibitors of HAT activity, such as curcumin and C646, have been shown to reduce histone acetylation and downregulate pain-related factors such as BDNF and COX-2 (16, 17). The interaction between NGF and epigenetic modifications has been previously documented. NGF has been reported to influence DNA methylation patterns and histone acetylation during neurite outgrowth by modulating the expression of DNA methyltransferases and histone deacetylases (18, 19). Specifically, NGF-induced changes in histone acetylation affect the expression of various pain-related genes, highlighting the significance of NGF in the epigenetic regulation of NP (20, 21). Our results align with these findings, as NGF-induced ERK activation was associated with increased acetylation of histone H3K56, a key modification linked to pain gene expression.

In contrast, other studies have reported differing effects of NGF on pain perception. For instance, some studies have demonstrated that intrathecal administration of NGF can alleviate hyperalgesia in neuropathic pain models (22, 23). In addition, the effects of histone deacetylase (HDAC) inhibitors on pain modulation are complex and context-dependent. HDAC inhibitors have been found to either reduce or exacerbate pain symptoms, depending on the specific gene targets and the nature of the pain model (24, 25). These discrepancies suggest that the role of NGF and histone modifications in pain may vary based on experimental conditions and specific pathways involved.

In summary, our research is the first to provide evidence that NGF contributes to neuropathic pain via an epigenetic mechanism within the CSF-CN, identifying this process as the NGF/ERK/Ac-H3K56 axis. We provide evidence that NGF is a regulator of histone acetylation and contributes to the development and maintenance of chronic pain. The identification of a new supraspinal regulatory site offers a new perspective on pain circuitry, highlighting mechanisms distinct from spinal or peripheral targets.

From a translation perspective, our findings provide opportunities for the pharmacological targeting of the CSF-CN or for the manipulation of the NGF/ERK axis to treat neuropathic pain. As described above, NGF-neutralizing antibodies, such as tanezumab, have shown promise in clinical trials, but the deep anatomical proximity of the ability to pharmacologically target the CSF-CN has many associated problems, particularly due to issues associated with surrounding anatomical and/or circulation (cerebrospinal fluid) roles. Future therapeutic paradigms may target supraspinal pain circuits using delivery systems, as these will be much more acceptable to patients for attempting to manipulate and regulate supraspinal functions ignoring any invasive nature to attempt to reach spinal circuitry. Furthermore, it will be important to determine if ERK inhibitors or epigenetic modulators can be developed safely for evaluation in clinical pharmacological therapies. Developing targeted, mechanism-based therapies for NP in humans will require a more comprehensive understanding of how NGF signaling is spatially and temporally regulated at the molecular level.

## 5 Conclusion

This study demonstrates that NGF plays a role in neuropathic pain via the ERK/Ac-H3K56 epigenetic pathway in the cerebrospinal fluid contacting nucleus (CSF-CN). To the best of our knowledge, this is the first report in the literature showing NGF-mediated epigenetic changes in this region above the spinal cord. These findings emphasize an additional epigenetic target for intervention beyond peripheral and/or spinal mechanisms, although further research is still required to confirm the precise HATs involved and to validate whether ERK inhibition directly mediates the observed H3K56 acetylation changes. In terms of clinical implication, NGF inhibitors, such as tanezumab, and ERK pathway inhibitors are potentially relevant; however, pharmacological targeting of the CSF-CN poses challenges due to its deep location within the CNS and its functional complexity. Future studies should focus on delivery and testing relevant translational models to examine the potential of targeting this pathway in human neuropathic pain.

## Data Availability

The raw data supporting the conclusions of this article will be made available by the authors, without undue reservation.
