# Supplementary figures and images for: NGF/ERK signaling-mediated epigenetic regulation of neuropathic pain in the cerebrospinal fluid-contacting nucleus
Source: Front Neurol. 2025 Oct 29;16:1641450. doi: 10.3389/fneur.2025.1641450 (PMC12614463; doi:10.3389/fneur.2025.1641450)

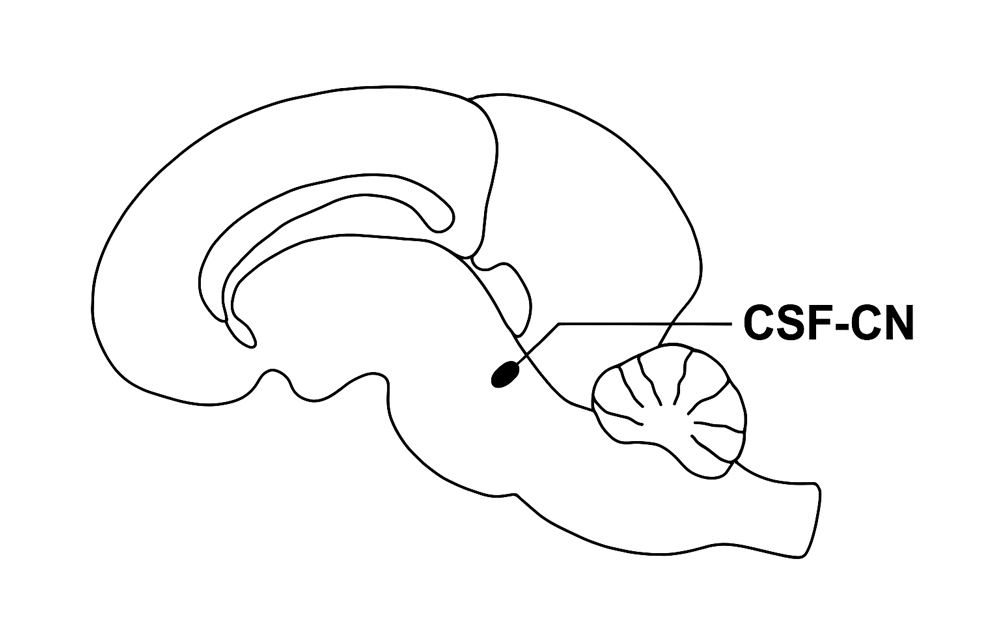

Supplement: Supplementary file 1 [file Image_1.TIF]
